# Clustering of *Cryptosporidium* species infections among sheep and cattle but not children in remote highland communities of Madagascar

**DOI:** 10.1186/s13071-022-05434-0

**Published:** 2022-08-28

**Authors:** Ralf Krumkamp, Franz J. Conraths, Simone Caccio, Gereon Schares, Benedikt Hogan, Doris Winter, Anna Jaeger, Sophia Melhem, Njari Rakotozandrindrainy, Jürgen May, Raphael Rakotozandrindrainy, Daniel Eibach

**Affiliations:** 1 Department of Infectious Disease Epidemiology, Bernhard Nocht Institute for Tropical Medicine, 20359 Hamburg, Germany; 2 German Center for Infection Research (DZIF), Partner Site Hamburg—Lübeck—Borstel—Riems, Hamburg, Germany; 3 Institute of Epidemiology, Friedrich-Loeffler-Institut—Federal Research Institute for Animal Health, 17493 Greifswald-Insel Riems, Germany; 4 Unit of Foodborne and Neglected Parasites, Istituto Superiore Di Sanità, Rome, Italy; 5 University of Antananarivo, Antananarivo, Madagascar; 6 Tropical Medicine II, University Medical Centre Hamburg-Eppendorf, 20151 Hamburg, Germany

**Keywords:** *Cryptosporidium*, Disease transmission, Madagascar, Rural area, Cluster analysis

## Abstract

**Background:**

The aim of this study was to identify local transmission patterns of *Cryptosporidium* spp. infections among livestock and humans in four extremely rural and remote highland communities in Madagascar.

**Methods:**

In this cross-sectional study, households were randomly sampled throughout a 1-year study period, with one feces sample collected from each child (≤ 5 years old), sheep and cattle. *Cryptosporidium* spp. were identified using a nested PCR assay targeting the* 18S* ribosomal RNA gene. All samples positive for *Cryptosporidium hominis* were further subtyped by sequencing the 60-kDa glycoprotein gene (*gp60*). Spatial clustering methods were applied to analyze potential transmission patterns.

**Results:**

In total, 252 households participated in the study, and samples from 197 children, 862 cattle and 334 sheep were collected and included in the study. Of the samples collected, 11 (5.6%) from children, 30 (3.5%) from cattle and 42 (12.6%) from sheep tested positive for *Cryptosporidium* spp. Very little overlap in the species distribution between human and animal infections was found. Global (overall) and local (spatially defined) clustering was observed for *Cryptosporidium* spp. infections in sheep and for *Cryptosporidium xiaoi/bovis* infections among sheep and cattle.

**Discussion:**

The results of this analysis do not support the occurrence of defined disease outbreaks, rather they point to a continuous series of transmission events that are spatially aggregated. Despite the close coexistence between humans, sheep and cattle in the study area, mutual transmission was not observed. Hence, the study underlines the importance of sustained sanitation and hygiene measures to prevent cryptosporidiosis transmission among infants, since asymptomatic children serve as an infection reservoir. Similarly, the study highlights the importance of improving hygiene to reduce the transmission of *Cryptosporidium* spp. in livestock, an infection with serious consequences, especially in newborn calves.

**Graphical Abstract:**

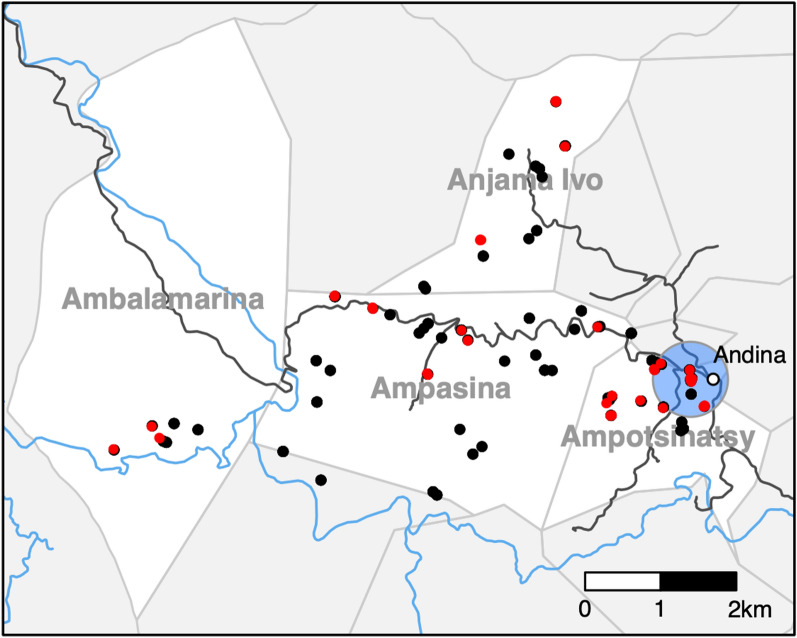

**Supplementary Information:**

The online version contains supplementary material available at 10.1186/s13071-022-05434-0.

## Introduction

Cryptosporidiosis has been identified as one of the main causes for childhood diarrhea in sub-Saharan Africa, with an estimated 2.9 million cases and 48,000 deaths annually [1]. Infections are associated with growth deficits and cognitive impairment [2, 3]. While diarrheal disease caused by *Cryptosporidium* spp. is the second most common cause of death [4], a large number of asymptomatic or mild infections are not diagnosed and remain undetected in the community [5]. These asymptomatic cases could serve as an infection reservoir for younger children, who are more likely to develop diarrheal disease [6]. It has been suggested that transmission in sub-Saharan Africa occurs primarily anthroponotically (i.e. human-to-human) within neighborhoods, while animals do not seem to contribute substantially to infections in humans [5]. Likewise, *Cryptosporidium* in ruminant livestock is clinically significant, with a wide spectrum of signs that range from asymptomatic infection to serious complications. In particular, cryptosporidiosis in calves is considered to be an important veterinary disease resulting in increased morbidity and mortality, as well as consequent production losses [7]. Because of the environmentally stable oocysts and the low infectious dose, cryptosporidiosis in cattle is difficult to control and the infection can be transmitted very quickly to susceptible hosts [8].

Madagascar is not only home to unique flora and fauna, but also supports an exceptional diversity of *Cryptosporidium* species (spp.). Studies have demonstrated high prevalence of *Cryptosporidium suis* [9], *Cryptosporidium xiaoi/bovis* and *Cryptosporidium* *ryanae* [5] in cattle, with a surprising absence of *Cryptosporidium parvum* and *Cryptosporidium andersoni*, which are commonly observed in livestock in many other countries. In humans, *Cryptosporidium hominis* [5, 10] and *Cryptosporidium meleagridis* [5] are the prevalent species. Inaccessibility and remoteness of large parts of the island, a large livestock population predominated by zebu cattle and extreme poverty with poor sanitation facilities might favor this specific epidemiological situation in both humans and animals. However, the transmission dynamics among different livestock species, among humans and between animals and humans in such remote settings are entirely unclear [11].

This study therefore aimed to identify local transmission patterns of *Cryptosporidium* spp. infections among sheep, cattle and humans in four extremely remote highland communities (locally referred to as “fokontanies”) in Madagascar over a 1-year period.

## Methods

### Study site and study design

**Fig. 1:**
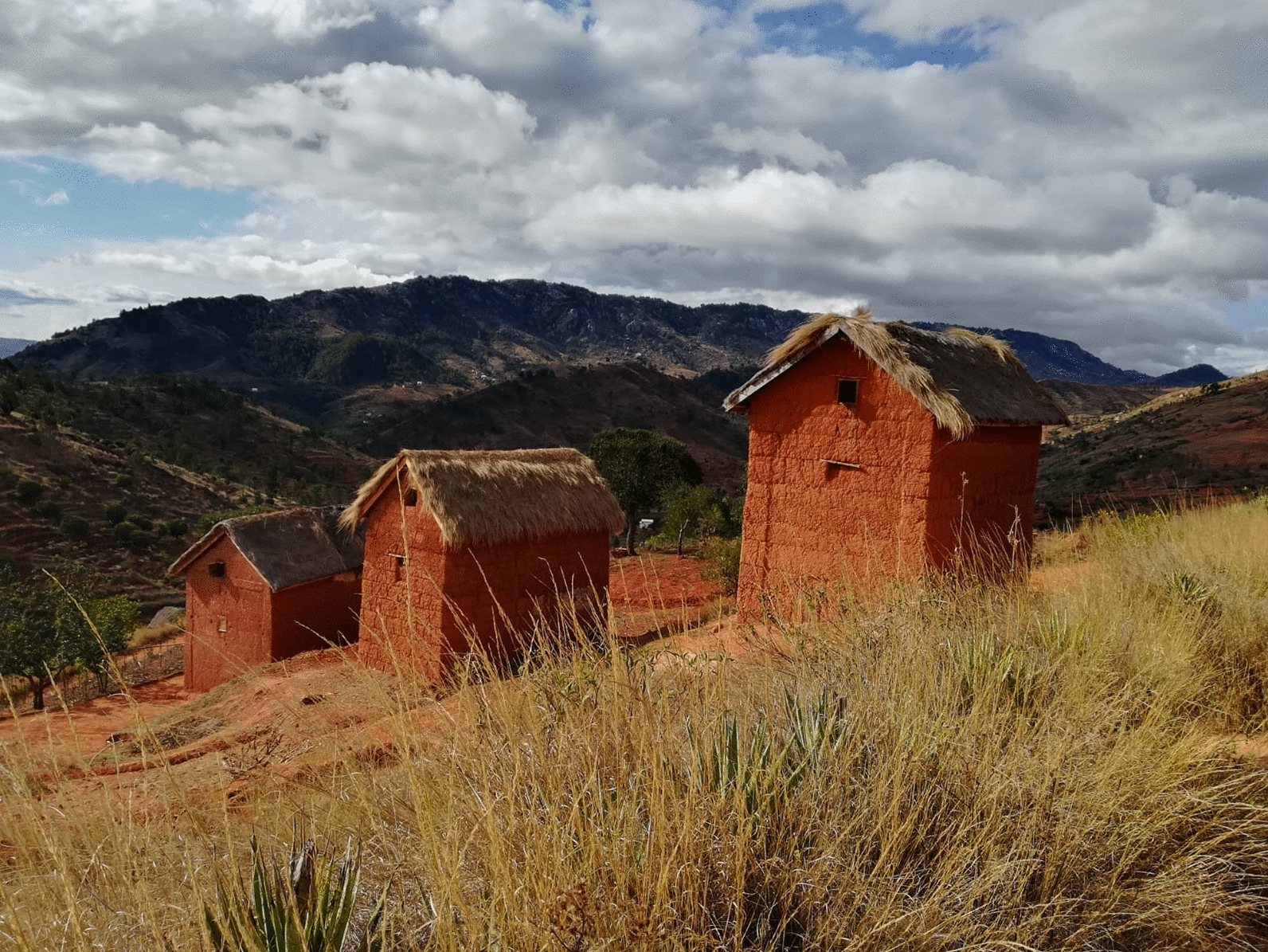
Typical housing conditions within the study area in the Ambositra region in Madagascar. Picture was provided by Daniel Eibach

All households within the communities of Ambalamarina, Ampasina, Anjama Ivo and Ampotsinatsy, all located in the Ambositra District of the Amoron’i Mania Region of Madagascar, were considered for inclusion in this cross-sectional study. The study area covered an area of about 34 km^2^ and is located in the highlands of Madagascar, approximately 150 km south of the capital Antananarivo. The study region is extremely remote and rural; communities have rudimentary road connections and households are primarily accessible by footpaths. Most community members work in farming, partly subsistence farming, or animal husbandry. Houses are typically set up in small groups, distributed throughout the whole study area. Figure 1 provides a picture of typical housing conditions within the study area. Commonly, animals are kept in the basement of each house, and the living area is located on the second floor. Sheep are primarily kept indoors and cattle next to the house.

Households were randomly sampled throughout the 1-year study period, which ranged from May 2017 to April 2018. All households within the study area (*N* = 329) were registered to define the sampling frame. About six neighboring households were grouped together and each group assigned a unique random number. Household groups were ordered by their random number and visited consecutively at weekly intervals. Stool containers were provided to household members, and they were asked to collect individual fresh feces samples from all children (aged ≤ 5 years) as well as from all sheep and cattle owned by the households. Stool containers were collected by field workers within 48 h and transported to the local laboratory on the same day for immediate processing. During the household visits participants were asked about their living conditions and the demographics of the people living in the households. The geographical coordinates of each household were also recorded.

### Detection and identification of *Cryptosporidium *spp.

DNA was extracted from a 250-μl aliquot of each stool/fecal sample using the Qiagen DNeasy PowerSoil Kit (Qiagen, Hilden, Germany) according to the manufacturer’s instructions. Samples were not pre-processed before DNA extraction. *Cryptosporidium* spp. were identified using a nested PCR assay protocol for the amplification and sequencing of the* 18S* ribosomal RNA (rRNA) gene, as published previously [12]. Classification to the species level was conducted by an alignment to reference sequences retrieved from the National Center for Biotechnology Information (NCBI) GenBank. The analyzed segment of the* 18S* rRNA gene did not allow *C. xiaoi* and *C. bovis* to be differentiated. All samples positive for *C. hominis* were further subtyped by sequencing a 850-bp fragment of the 60-kDa glycoprotein gene (*gp60*) using a nested PCR as previously described [13, 14]. The assignment to *C. hominis* subtypes was performed according to the number of trinucleotide repeats (TCA, TCG or TCT) coding for the amino acid serine, as described elsewhere [15].

### Data analysis

Categorical variables were described using the frequency and percentage, and continuous variables were described with the median and interquartile range (IQR). The occurrence of *Cryptosporidium* spp. in humans, cattle and sheep was analyzed over time by accumulating the number of cases on the species level per month for each host group. Monthly average precipitation data were obtained from www.worldweatheronline.com to assess seasonal patterns. The occurrence of *Cryptosporidium* spp. within households was analyzed by comparing infections among household members and animals kept by the household. The variables used in the analysis did not contain any missing values.

Global spatial clustering was analyzed by means of the *k*-nearest neighbor (*k*NN) test for case–control data, as proposed by Cuzick and Edwards [16]. This test sums up the number of cases among a predefined number (*k*) of nearest case and control neighbors and compares this to the expected number under the random labeling hypothesis. The test was based on 1000 simulation runs with nearest neighbor groups of *k* = 10, 20, 30, 40 and 50 subjects. To localize potential case clusters, we applied the spatial scan test of Kulldorf for case–control point data [17]. The maximum radius of a cluster was set to 500 m, and the *P*-value was computed based on 500 simulations. All clusters with a *P*-value < 0.1 were reported in the analysis. The cluster analyses were applied to the human, cattle and sheep data as well as to cattle and sheep data considering *C. xiaoi/bovis* infections only. Due to the exploratory nature of the analysis, no statistical significance levels were defined. All analyses were conducted with the statistical software R version 4.0.3 (R Foundation for Statistical Computing, Vienna, Austri﻿a﻿) using the packages *spatstat* (2.1–0) for ANN analysis, *smacpod* (2.1.1) to calculate cluster scan statistics and *sp* (1.4–5) and *ragdal* (1.5–23) to draw spatial maps. The shape files used to draw the maps were taken from Humanitarian Data Exchange (HDX) website (https://data.humdata.org). The study data used for the analysis can be found in the Supplementary Information (Additional file 1: Table S1). For data protection reasons, the geographical coordinates of the households cannot be made publicly available.

## Results

Of the 329 registered households in the study area, 252 (76.5%, 252/329) participated in the study. The median number of persons living in the households included in the study was eight (IQR: 6–9). Houses were primarily constructed of mud (75.3%, 190/252) or brick (23.8%, 60/252); in two houses the material was unknown. Twelve (4.7%, 12/252) households reported having electricity, and almost all households obtained drinking water from boreholes (98.8%, 249/252). Pit latrines were the main toilet type (67.5%, 170/252), although 71 (28.2%, 71/252) households reported defecating in the open. All respondents lived with animals in their homes. The following animals were kept by families: chickens (97.2%, 245/252), cattle (88.1%, 222/252), pigs (42.9%, 108/252,), sheep (30.6%, 77/252), dogs (27.0%, 68/252), cats (7.1%, 18/252) and goats (2.0%, 5/252). The median number of animals kept per household was 11 (IQR: 10–17) chickens, four (IQR: 2–5) cattle, two (IQR: 2–4) pigs, four (IQR: 3–6) sheep and two (IQR: 1–3) goats.

From the 252 households participating in the study, 232 children aged ≤ 5 years, 889 cattle and 353 sheep were included in the study, but samples could only be collected from 197 (84.9%, 197/232) children, 862 (97.0%, 862/889) cattle and 334 (94.6%, 334/353) sheep. Table 1 summarizes the characteristics by study subjects. A median number of five (IQR: 3–7) samples per household were collected. Samples were collected from children in 133 households (52.8%, 133/252), from cattle in 222 households (88.1%, 222/252) and from sheep in 75 households (29.8%, 75/252). A total of 11 human samples (5.6%, 11/197), 30 cattle samples (3.5%, 30/862) and 42 sheep samples (12.6%, 42/334) tested positive for *Cryptosporidium* spp. The highest percentage of human infections was recorded in Anjama Ivo community (9.1%, 4/44), and the highest percentage of cattle and sheep infections were recorded in Ampotsinatsy community (6.4, 14/218 and 18.6, 22/118, respectively). None of the children who tested positive for *Cryptosporidium* spp. reported diarrhea during the last 24 h, and the age of children who tested positive and negative for *Cryptosporidium* spp. was comparable (in both, median = 3 years, IQR: 1–3). Infections in humans were due to *C. hominis* (63.6%, 7/11), *C. meleagridis* (18.2%, 2/11) and *Cryptosporidium viatorum* (18.2%, 2/11). The predominant* Cryptosporidium* species detected in cattle was *C. xiaoi*/*bovis* (73.3%, 22/30), followed by *C. ryanae* (23.3%, 7/30) and *C. viatorum* (3.3%, 1/30), and all infections in sheep were due to *C. xiaoi*/*bovis* (100%, 42/42). Human infections with *C. hominis* were all identified as *gp60* subtype IbA10G2. As indicated by the overall species distribution, only little overlap between human and animal infections was observed, i.e. *C. viatorum* was the only *Cryptosporidium* species observed in both humans and cattle*.*

**Table 1:** Characteristics of the 252 sampled households by study subject group

Characteristics	Study subject group		
	Children (*N* = 197)	Cattle (*N* = 862)	Sheep (*N* = 334)
Female sex,* n* (%)	107 (54.3)	NA	NA
Age in years, median (IQR)	3 (1–3)	NA	NA
Diarrhea during the last 24 h,* n* (%)	8 (4.1)	NA	NA
Households with samples,* n*/*N* (%)	133/252 (52.8)	222/252 (88.1)	75/252 (29.8)
Samples per household, median (IQR)	2 (1–2)	3 (2–5)	4 (3–6)
*Cryptosporidium* spp.,* n*/*N* (%)	11/197 (5.6)	30/862 (3.5)	42/334 (12.6)
* C. hominis*	7/11 (63.6)	0 /30(0)	0/42 (0)
* C. meleagridis*	2/11 (18.2)	0/30 (0)	0/42 (0)
* C. ryanae*	0/11 (0)	7/30 (23.3)	0/42 (0)
* C. viatorum*	2/11 (18.2)	1/30 (3.3)	0/42 (0)
* C. xiaoi/bovis*	0/11 (0)	22/30 (73.3)	42/42 (100)
*gp60* subtype,* n*/*N* (%)			
* C. hominis IbA10G2*	6/6 (100)	NA	NA
*Cryptosporidium* spp. infection per community,* n*/*N* (%)			
Ambalamarina	1/18 (5.6)	2/76 (2.6)	4/29 (13.8)4
Ampasina	3/86 (3.5)	11/338 (3.3)	9/144 (6.2)
Ampotsinatsy	3/49 (6.1)	14/218 (6.4)	22/118 (18.6)
Anjama Ivo	4/44 (9.1)	3/230 (1.3)	7/43 (16.3)

*gp60* 60-kDa glycoprotein, *IQR* interquartile range, *NA* not assessed

**Fig. 2:**
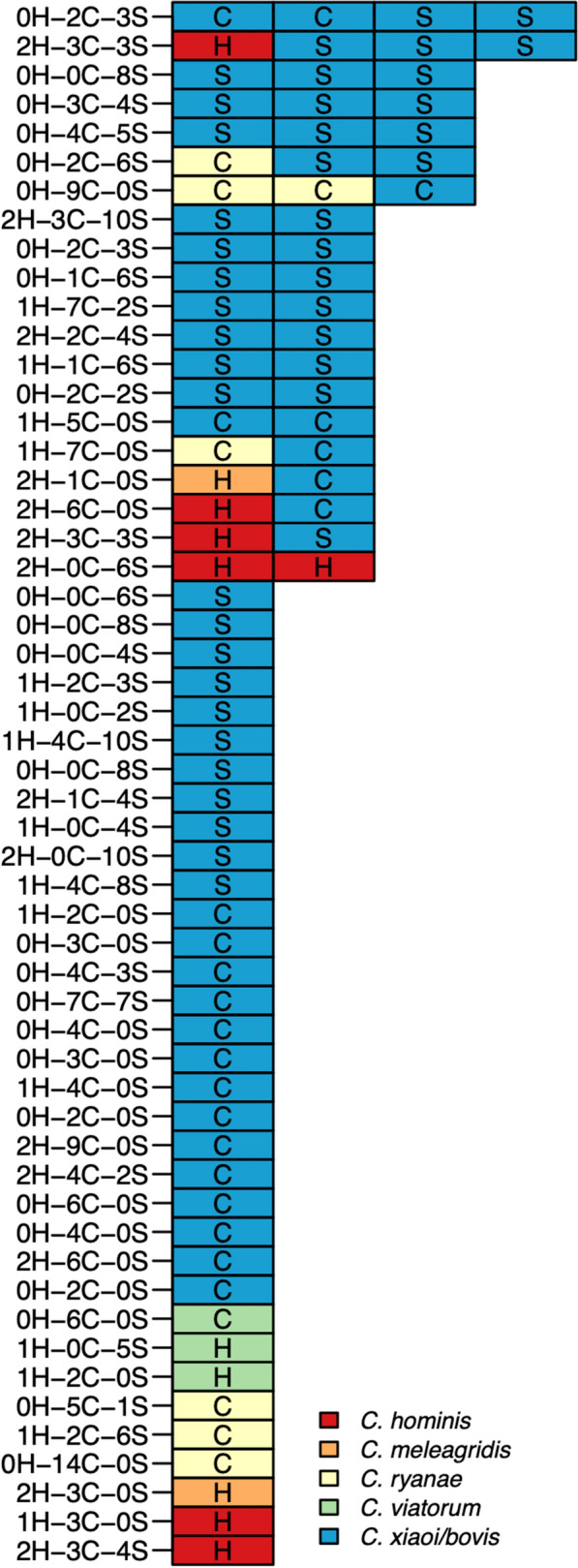
Number of cases of *Cryptosporidium* spp. infections per affected household, stratified by species or taxa. Each row of boxes represents a household, and each box in that row represents a sample that tested positive for *Cryptosporidium* infection. The category of infected subject is indicated by uppercase letters within boxes (H, humans; C, cattle; S, sheep), and the diagnosed *Cryptosporidium* species or taxon is indicated by color coding (bottom right). Text and numbers to the left of the rows of boxes indicate the number of samples taken for each category of subjects (H, C, S) within the respective household

A total of 83 infections were detected, all within 54 (21.4%, 54/252) of participating households. The infected subjects and responsible species or taxa per household are illustrated in Fig. 2. In 34 households (63.0%, 34/54) a single *Cryptosporidium* species was recorded, in 13 households (24.1%, 13/54) two *Cryptosporidium* spp. were detected and in seven households (13.0%, 7/54) three or more *Cryptosporidium* spp. were detected. Human infections were detected in 10 households (18.5%, 10/54), and in only in one household two human infections were diagnosed, both caused by *C. hominis* IbA10G2*.* No overlap in *Cryptosporidium* spp. between humans and animals on the household level was detected. In two households, infections in both cattle and sheep were observed, and in one of these, the same species group (*C. xiaoi/bovis*) was detected.

**Fig. 3:**
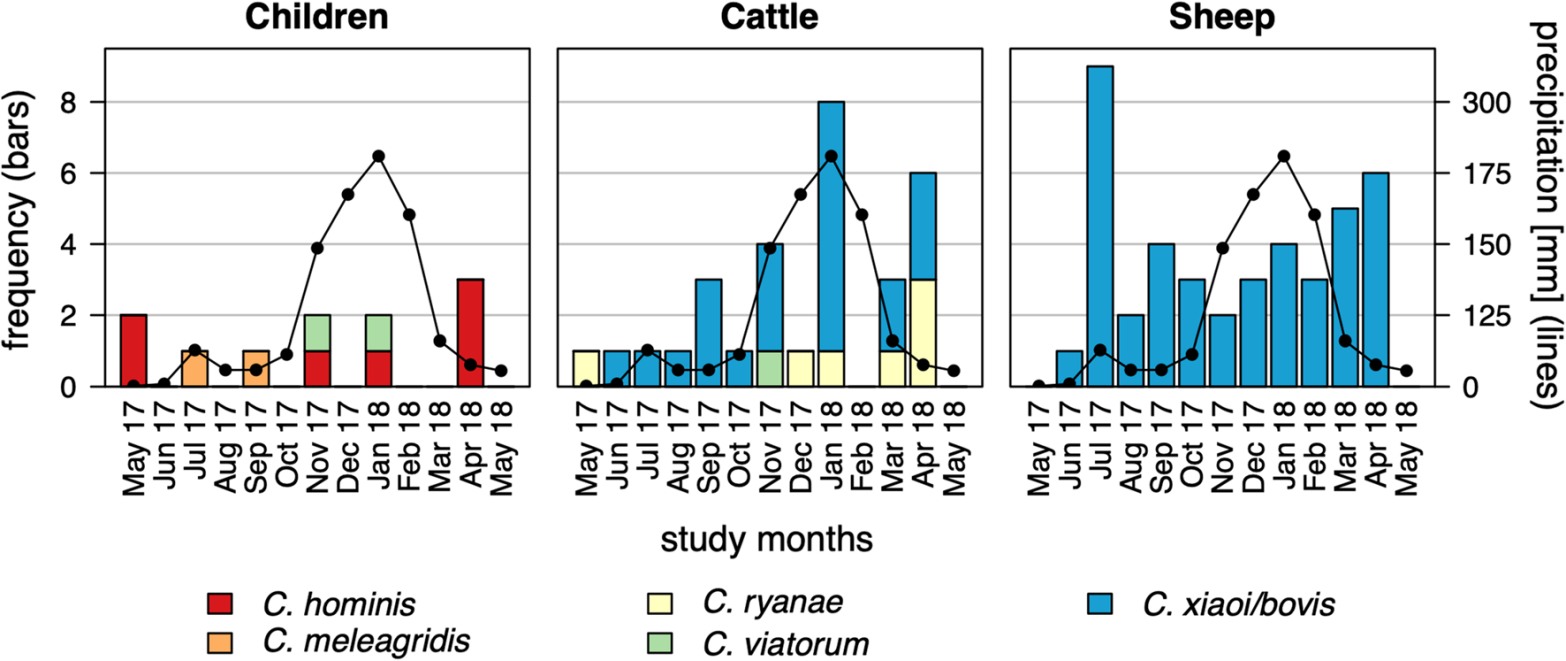
Seasonal occurrence of *Cryptosporidium* spp. infections in children, cattle and sheep in the Ambositra region, Madagascar. Precipitation data are shown by a black line

Seasonal patterns of *Cryptosporidium* spp. detection on the species level for humans, cattle and sheep are shown in Fig. 3, along with the average monthly precipitation. Infections in humans occurred sporadically throughout the study year. Infections in cattle showed a peak in January 2018, with eight cases, which correlated with the highest amount of rainfall. However, a clear correlation with monthly precipitation was not evident. Infections in sheep occurred throughout the year without any obvious seasonal pattern.

**Fig. 4:**
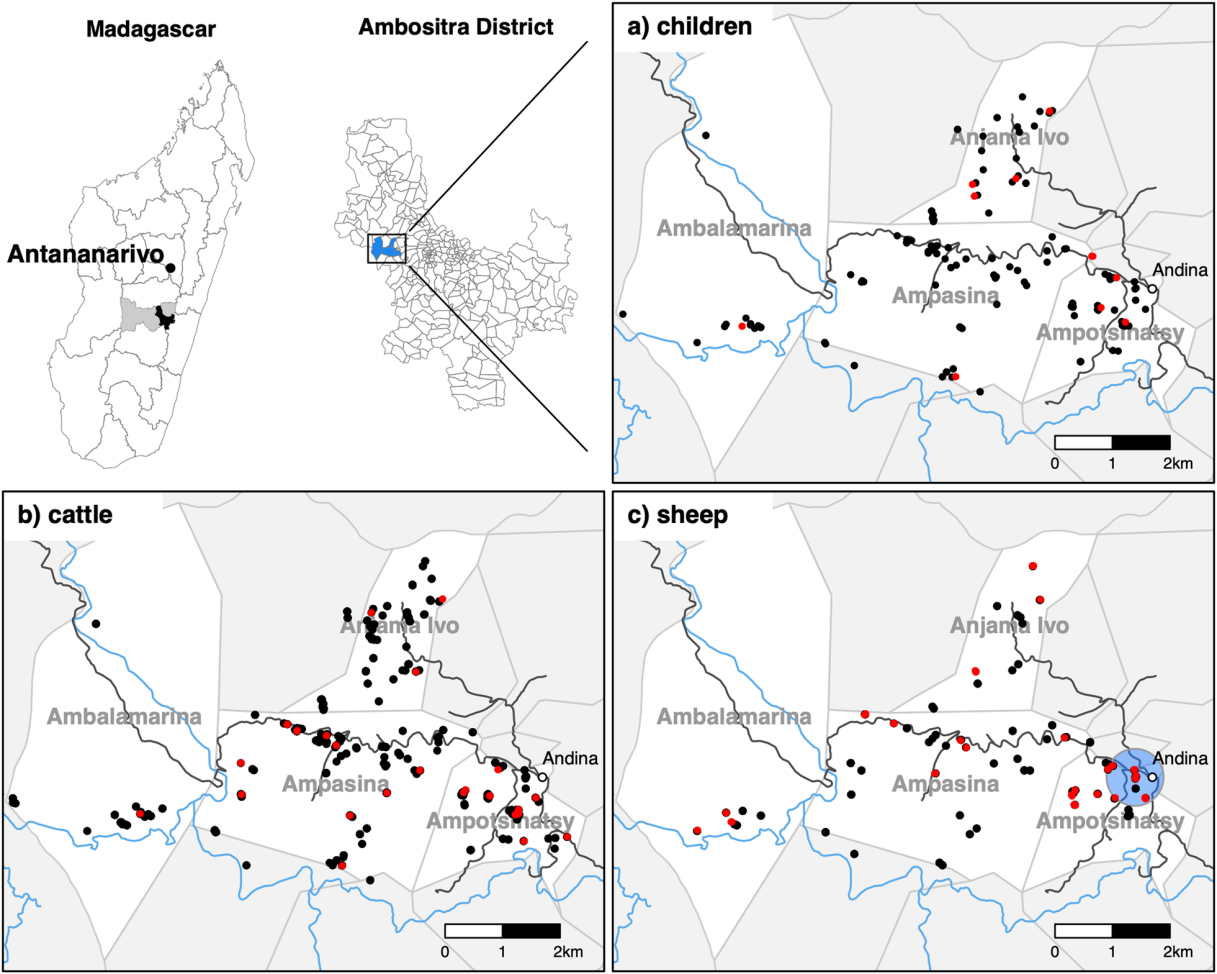
Geographical distribution of study subjects in the four study communities (marked in blue on the Ambositra District map) in the Ambositra District (marked in black on the map of Madagascar) of the Amoron’i Mania region (marked in gray on the map of Madagascar), Madagascar. Red dots on the case maps (**a**–**c**) indicate subjects that tested positive for *Cryptosporidium* spp. infection and black dots indicate subjects that tested negative. The blue circle on the sheep map (**c**) indicates a cluster identified by spatial scan statistics

Figure 4a–c shows the geographical distribution of *Cryptosporidium* spp. infections in children, cattle and sheep. Global clustering of infections was analyzed with the Cuzick and Edwards’ test [16] for the study subject groups (Table 2). Test statistics for different numbers of nearest neighbors in humans and cattle were all ≥ 0.10 and did not suggest clustering of infections. However, infections in sheep with *k*NN ≤ 50 led to *P-*values ≤ 0.04, indicating a generally larger number of cases in the proximity of other cases. The lowest *P*-values were observed when the analysis was limited to *C. xiaoi/bovis* infections in cattle and sheep. The low *P*-values in all *k*NN-groups indicate that infections of cattle and sheep with *C. xiaoi/bovis* occurred in clusters.

**Table 2:** Global clustering analysis of *Cryptosporidium* spp. infections

Number of NN	Study subject group			
	Children	Cattle	Sheep	*C. xiaoi/bovis* in cattle and sheep
10	0.33	0.12	0.01	< 0.01
20	0.33	0.49	0.03	< 0.01
30	0.09	0.29	0.04	0.01
40	0.25	0.16	0.02	< 0.01
50	0.24	0.15	0.04	< 0.01
100	0.62	0.10	0.76	< 0.01

The table lists *P*-values obtained using the Cuzick and Edwards’ test considering different numbers in *k*-nearest neighbor (NN) test (*k*NN) for *Cryptosporidium* spp. infections in the different groups of study subjects and for *C. xiaoi/bovis* infections in cattle and sheep

Global cluster methods are designed to identify overall clustering within a population. To locate potential clusters, we applied scan statistics for case–control data. A cluster (*P* = 0.05) was identified for sheep, which was located west of Andina in the Ampotsinatsy fokontany. This cluster is indicated with a blue circle on the map showing sheep data in Fig. 4c. The circular cluster had a radius of 496 m, was comprised of 45 sheep from nine households and corresponded to the main market center of the entire area. Of these sheep, 14 (31.1%, 14/45) were *Cryptosporidium* spp. positive and belonged to eight households. The cases did not accumulate over time. Scan statistics were also applied to both cattle and sheep considering *C. xiaoi/bovis* infections only. A case cluster was identified (*P* = 0.002) within the same area as the sheep cluster described above, comprising the 45 sheep from the first cluster along with 38 cattle. This cluster consisted of 13 households. Three of these cattle (7.9%, 3/38) tested positive for *Cryptosporidium* spp. and belonged to two households. In one household, two sheep and two cattle tested positive at the time of sampling (January 2018).

## Discussion

The results of this study provide insights into the local transmission dynamics of *Cryptosporidium* spp. in rural communities in the highlands of Madagascar, where animals and humans live in close proximity. They reveal geospatial clustering of *Cryptosporidium* spp. infections among sheep and among sheep and cattle in remote highland communities of Madagascar. Since infections do not aggregate over time, our data do not support the occurrence of defined disease outbreaks but rather point to spatially aggregated series of transmission events, i.e. to an endemic situation of cryptosporidiosis in the tested animal populations. A local *Cryptosporidium* cluster was detected in the study area around the town of Andina. Andina is the largest village and main trading center of the study area, with a population of 25,650, and holds weekly livestock markets where animals from the surrounding communities are traded. Thus, this relatively higher animal density with different livestock species may result in higher odds that humans and animals transmit and/or contract *Cryptosporidium* infection, leading to this geographical cluster.

Bodager et al*.* [9] conducted a similar study in which they examined the distribution of *Cryptosporidium* spp. in humans, domestic animals, peridomestic rodents and wildlife in and around Ranomafana National Park in Madagascar, a rural area located about 100 km south of our study side. The predominant *Cryptosporidium* species identified by these researchers was *C. suis*, which they detected in cattle (*n/N* = 17/62), pigs (*n/N* = 3/17) and also humans (*n/N* = 1/120). In contrast, we did not detect *C. suis* in our study. The second most common species detected by Bodager et al*.* [9] was *C. meleagridis*, which they found in rodents (*n/N* = 7/48). However, in our study we did not sample rodents. These two studies (Bodager et al*.* [9] and the present study) differ in some aspects of methodology, such as the subjects studied, the number of samples collected and the time periods in which the studies were conducted. However, it is interesting to note that the detected *Cryptosporidium* spp. differ between the studies, which may indicate that less connected rural communities have a unique species distribution. In addition, the environmental and living conditions at the two study sites differ, possibly indicating that predominant transmission routes have influenced the observed species distribution.

It is of interest that in our study only *C. xiaoi/bovis* was detected in sheep, while cattle were found to be infected with *C. ryanae, C. viatorum* or *C*. *xiaoi/bovis*. *Cryptosporidium* *xiaoi* has been reported as the dominant species in small ruminants outside of Europe, and it is believed to have minimal, if any, zoonotic potential [18]. Four *Cryptosporidium* spp., i.e. *C. parvum*, *C. bovis*, *C. ryanae* and *C. andersoni,* are common in cattle, but only *C. parvum* seems to be associated with clinical disease in neonatal calves [8]. Furthermore, the prevalence of these species is known to vary with the age of the animal, with *C. parvum* being predominant in suckling calves, *C. bovis* and *C. ryanae* in post-weaned calves and *C. andersoni* in adults [19]. As data on the age of the sheep and cattle examined in this study were not available, we cannot exclude that the absence of *C. parvum* and *C. andersoni* simply reflects a lack of very young or adult animals among those animals we could sample. However, to the best of our knowledge, *C. parvum* has been reported only from one pig and one human sample in Madagascar [9, 10], suggesting a low prevalence of this pathogen.

The finding of *C. viatorum* in cattle was unexpected, given that this species is considered to be a human-specific pathogen [20]. However, researchers in Australia and China identified *C. viatorum* in rats, suggesting zoonotic potential [21, 22]. Therefore, we cannot exclude that the presence of *C. viatorum* in cattle could be the result of the mechanical passage of oocysts rather than a true infection.

The observed distribution of *Cryptosporidium* spp. among infected children was very different from that among animals. Not surprisingly, the majority of cases in children were due to *C. hominis*, further confirming that human-to-human transmission of cryptosporidiosis predominates in the African continent [3, 7, 24]. However, subtyping at the *gp60* gene locus revealed subtype IbA10G2 in these children, reinforcing previous data from isolates collected in the capital Antananarivo, as well as in the context of a large epidemiologic study in Africa that included Madagascar [5]. This subtype is the most common *C. hominis* subtype in Europe and high-income countries [23, 24], but is very uncommon in other African countries [5, 11, 25], suggesting an epidemiological situation unique to Madagascar. It should be noted, however, that children were infected also with *C. viatorum* (2 cases) and *C. meleagridis* (2 cases). This suggests that zoonotic transmission involving rodents and poultry cannot be ruled out, particularly in a context of poor housing and living conditions.

All children who tested positive for *Cryptosporidium* spp. in the present study were asymptomatic, supporting a previous hypothesis that a number of cryptosporidiosis cases are constantly circulating in communities, serving as a silent reservoir for younger children aged < 2 years [6]. The latter children are more likely to develop symptomatic diarrheal disease and suffer further consequences in terms of growth faltering and cognitive outcomes [2, 3]. A similar situation has long been known in young animals, in particular calves and lambs infected with *C. parvum* [8, 26].

Our study has methodological limitations, which should be considered when interpreting the results. We could not include the age of the animals in our analysis, but as already mentioned, it is known that newborn calves and lambs are particularly susceptible to* Cryptosporidium* infections. Data on animal age would allow us to distinguish between infections in young and adult animals and to better understand the observed species distribution. Likewise, possible seasonal variations in infections due to calving cannot be assessed, but for the study area this effect can be considered minor, since calves are born throughout the year. In addition, we do not have data on* Cryptosporidium* infections in pigs and chickens, two animals commonly kept by households in the study area. Because their infection data is lacking, we cannot make a comprehensive assessment of transmission clusters. In their study in rural southern Madagascar, Bodager et al. found *C. suis* and *C. parvum* in pigs; these two species were not observed in our study [9]. Data on* Cryptosporidium* infection in poultry show that *C. meleagridis* is one of the most relevant species, including infections in African countries, and zoonotic transmission has been suggested [11]. Two children in our study were also infected with *C. meleagridis*. However, it should be noted that this is the third most commonly reported species in humans [27], thus human-to-human transmission is also likely. We randomly sampled all subjects over a 1-year period, regardless of acute gastrointestinal symptoms. However, applying this sampling approach may have resulted in smaller *Cryptosporidium* spp. clusters or outbreaks being missed that may have been detectable in a search for acute cases. With the current study design, infection reservoirs and the temporal and spatial clustering of infections in human and animal populations can be assessed.

## Conclusion

This study strengthened current understanding of the particular epidemiology of *Cryptosporidium* spp. infections in Madagascar. The data show that infections do not aggregate over time, thereby suggesting no occurrence of defined and larger disease outbreaks. Rather, the data point to continuous series of transmission events that are spatially aggregated. To fully understand transmission dynamics, infection data from all relevant human-animal contacts must be evaluated, which was not possible in our study. However, the data presented here underline the importance of sustained sanitation and hygiene measures to prevent cryptosporidiosis transmission among infants, since asymptomatic children serve as an important infection reservoir. Similarly, the study highlights the value of improving hygiene to reduce the transmission of *Cryptosporidium* spp. in livestock, an infection with serious consequences, especially in newborn calves.

## Supplementary Information

**Additional file 1: Table S1.** Study data file.

## Data Availability

The primary data set used for the study is available in the supplementary information. For data protection reasons, the geographic coordinates of the households cannot be provided.

## Abbreviations

IQR: Interquartile range
*k*NN: *k*-Nearest neighbor

## References

[1] Sow SO, Muhsen K, Nasrin D, Blackwelder WC, Wu Y, Farag TH (2016). The burden of *cryptosporidium* diarrheal disease among children < 24 months of age in moderate/high mortality regions of sub-saharan Africa and South Asia, utilizing data from the global enteric multicenter study (GEMS). PLoS Negl Trop Dis.

[2] Khalil IA, Troeger C, Rao PC, Blacker BF, Brown A, Brewer TG (2018). Morbidity, mortality, and long-term consequences associated with diarrhoea from *Cryptosporidium* infection in children younger than 5 years: a meta-analyses study. Lancet Glob Health.

[3] Korpe PS, Haque R, Gilchrist C, Valencia C, Niu F, Lu M (2016). Natural history of *Cryptosporidiosis* in a longitudinal study of slum-dwelling bangladeshi children: association with severe malnutrition. PLoS Negl Trop Dis.

[4] Wang H, Naghavi M, Allen C, Barber RM, Bhutta ZA, Carter A (2016). Global, regional, and national life expectancy, all-cause mortality, and cause-specific mortality for 249 causes of death, 1980–2015: a systematic analysis for the global burden of disease study 2015. Lancet.

[5] Krumkamp R, Aldrich C, Maiga-Ascofare O, Mbwana J, Rakotozandrindrainy N, Borrmann S (2021). Transmission of *Cryptosporidium* species among human and animal local contact networks in sub-saharan Africa: A Multicountry study. Clin Infect Dis.

[6] Korpe PS (2020). The silent reservoir of *Cryptosporidiosis*. Clin Infect Dis.

[7] Robertson LJ, Johansen ØH, Kifleyohannes T, Efunshile AM, Terefe G (2020). *Cryptosporidium* infections in Africa—how important is zoonotic transmission? a review of the evidence. Front Vet Sci.

[8] Thomson S, Hamilton CA, Hope JC, Katzer F, Mabbott NA, Morrison LJ (2017). Bovine cryptosporidiosis: impact, host-parasite interaction and control strategies. Vet Res.

[9] Bodager JR, Parsons MB, Wright PC, Rasambainarivo F, Roellig D, Xiao L (2015). Complex epidemiology and zoonotic potential for *Cryptosporidium suis* in rural Madagascar. Vet Parasitol.

[10] Areeshi M, Dove W, Papaventsis D, Gatei W, Combe P, Grosjean P (2008). *Cryptosporidium* species causing acute diarrhoea in children in antananarivo Madagascar. Ann Trop Med Parasitol.

[11] Squire SA, Ryan U (2017). *Cryptosporidium* and *Giardia* in Africa: current and future challenges. Parasit Vectors.

[12] Xiao L, Morgan UM, Limor J, Escalante A, Arrowood M, Shulaw W (1999). Genetic diversity within *Cryptosporidium parvum* and related *Cryptosporidium* species. Appl Environ Microbiol.

[13] Alves M, Xiao L, Sulaiman I, Lal AA, Matos O, Antunes F (2003). Subgenotype analysis of *Cryptosporidium* isolates from humans, cattle, and zoo ruminants in Portugal. J Clin Microbiol.

[14] Strong WB, Gut J, Nelson RG (2000). Cloning and sequence analysis of a highly polymorphic *Cryptosporidium parvum* gene encoding a 60-kiloDalton glycoprotein and characterization of its 15- and 45-kilodalton zoite surface antigen products. Infect Immun.

[15] Sulaiman IM, Hira PR, Zhou L, Al-Ali FM, Al-Shelahi FA, Shweiki HM (2005). Unique endemicity of *Cryptosporidiosis* in children in Kuwait. J Clin Microbiol.

[16] Cuzick J, Edwards R (1990). Spatial clustering for inhomogeneous populations. J R Stat Soc Ser B.

[17] Kulldorff M, Nagarwalla N (1995). Spatial disease clusters: detection and inference. Stat Med.

[18] Guo Y, Li N, Ryan U, Feng Y, Xiao L (2021). Small ruminants and zoonotic cryptosporidiosis. Parasitol Res.

[19] Díaz P, Navarro E, Remesar S, García-Dios D, Martínez-Calabuig N, Prieto A (2021). The age-related *Cryptosporidium* species distribution in asymptomatic cattle from North-Western Spain. Animals.

[20] Elwin K, Hadfield SJ, Robinson G, Crouch ND, Chalmers RM (2012). *Cryptosporidium*
*viatorum* n. sp (Apicomplexa: *Cryptosporidiidae*) among travellers returning to Great Britain from the Indian subcontinent, 2007–2011. Int J Parasitol.

[21] Koehler AV, Wang T, Haydon SR, Gasser RB (2018). *Cryptosporidium viatorum* from the native australian swamp rat rattus lutreolus—an emerging zoonotic pathogen?. Int J Parasitol Parasites Wildl.

[22] Zhao W, Zhou H, Huang Y, Xu L, Rao L, Wang S (2019). *Cryptosporidium* spp. in wild rats (*Rattus* spp.) from the Hainan Province, China: molecular detection, species/genotype identification and implications for public health. Int J Parasitol Parasites Wildl.

[23] Cacciò SM, Chalmers RM (2016). Human *Cryptosporidiosis* in Europe. Clin Microbiol Infect.

[24] Chalmers RM, Robinson G, Elwin K, Elson R (2019). Analysis of the *Cryptosporidium* spp. and gp60 subtypes linked to human outbreaks of cryptosporidiosis in England and Wales, 2009 to 2017. Parasit Vectors.

[25] Eibach D, Krumkamp R, Al-Emran HM, Sarpong N, Hagen RM, Adu-Sarkodie Y (2015). Molecular characterization of *Cryptosporidium* spp. among children in rural ghana. PLoS Negl Trop Dis.

[26] Holland RE (1990). Some infectious causes of diarrhea in young farm animals. Clin Microbiol Rev.

[27] Ryan U, Zahedi A, Feng Y, Xiao L (2021). An update on zoonotic *cryptosporidium* species and genotypes in humans. Animals.

